# Electroacupuncture Pretreatment Alleviates Cerebral Ischemic Injury Through α7 Nicotinic Acetylcholine Receptor-Mediated Phenotypic Conversion of Microglia

**DOI:** 10.3389/fncel.2019.00537

**Published:** 2019-12-06

**Authors:** Zhi Ma, Zengli Zhang, Fuhai Bai, Tao Jiang, Chaoying Yan, Qiang Wang

**Affiliations:** ^1^Department of Anesthesiology, Center for Brain Science, The First Affiliated Hospital of Xi’an Jiaotong University, Xi’an, China; ^2^Department of Anesthesiology, Xijing Hospital, Fourth Military Medical University, Xi’an, China

**Keywords:** stroke, electroacupuncture, α7 nicotinic acetylcholine receptor, microglia, inflammation

## Abstract

Electroacupuncture (EA) pretreatment alleviates cerebral ischemic injury through α7 nicotinic acetylcholine receptor (α7nAChR). We attempted to investigate whether the phenotypic conversion of microglia was involved in the therapeutic effect of EA pretreatment in cerebral ischemia through α7nAChR. Adult male Sprague–Dawley (SD) rats were subjected to middle cerebral artery occlusion (MCAO) after EA or α7nAChR agonist *N*-(3*R*)-1-azabicyclo[2.2.2]oct-3-yl-furo[2,3-*c*]pyridine-5-carboxamide hydrochloride (PHA-543,613 hydrochloride) and antagonist α-bungarotoxin (α-BGT) pretreatment. Primary microglia were subjected to drug pretreatment and oxygen-glucose deprivation (OGD). The expressions of the classical activated phenotype (M1) microglia markers induced nitric oxide synthase (iNOS), interleukin-1β (IL-1β), and cluster of differentiation 86 (CD86); the alternative activated phenotype (M2) microglia markers arginase-1 (Arg-1), transforming growth factor-β1 (TGF-β1), and cluster of differentiation 206 (CD206); and the pro-inflammatory cytokines tumor necrosis factor-α (TNF-α), interleukin-6 (IL-6), and anti-inflammatory cytokines interleukin-4 (IL-4) and interleukin-10 (IL-10) in the ischemic penumbra or in the supernatant of primary microglia were analyzed. The infarction volume and neurological scores were assessed 72 h after reperfusion. The cell viability and lactate dehydrogenase (LDH) release of neurons co-cultured with microglia were analyzed using cell counting kit-8 (CCK-8) and LDH release assays. EA pretreatment decreased the expressions of M1 markers (iNOS, IL-1β, and CD86) and pro-inflammatory cytokines (TNF-α and IL-6), whereas it increased the expressions of M2 markers (Arg-1, TGF-β1, and CD206) and anti-inflammatory cytokines (IL-4 and IL-10) by activating α7nAChR. EA pretreatment also significantly reduced the infarction volume and improved the neurological deficit. The activation of α7nAChR in microglia relieved the inflammatory response of primary microglia subjected to OGD and attenuated the injury of neurons co-cultured with microglia. In conclusion, EA pretreatment alleviates cerebral ischemic injury through α7nAChR-mediated phenotypic conversion of microglia, which may be a new mechanism for the EA pretreatment-induced neuroprotection against cerebral ischemia.

## Introduction

Stroke is one of the leading causes of death worldwide (Mozaffarian et al., 2016). High mortality and disability after stroke significantly increase the financial burden of the society and threaten public health. In response to ischemic stroke, microglia in the ischemic penumbra are excessively activated and show two different phenotypes in the early and late stages after ischemic stroke. The classical activated phenotype (M1) microglia are immediately activated after ischemic stroke and release robust inflammatory cytokines, such as interleukin-1β (IL-1β), tumor necrosis factor-α (TNF-α), and induced nitric oxide synthase (iNOS), which contribute to neuronal apoptosis and aggravate brain injury (Denes et al., 2007; Hu et al., 2012; DeGracia, 2013; Garden, 2013; Wang et al., 2013). At later time points after ischemic stroke, M1 phenotype microglia converted to the alternative activated phenotype (M2) microglia, which release anti-inflammatory cytokines and neurotrophic substances and contribute to repair of injury and neuroprotection (Kawabori and Yenari, 2015; Zhai et al., 2017). It may be a potential therapeutic target for the treatment of ischemic stroke to facilitate the phenotypic conversion of microglia from M1 to M2.

Electroacupuncture (EA), as a combination of traditional acupuncture and electrotherapy, not only inherits the benefits of traditional acupuncture but also integrates with the physiological effects of electric stimulation (Napadow et al., 2005). Previous studies have reported that EA effectively ameliorates TNF-α and interleukin-6 (IL-6) expressions in activated microglia (Liu et al., 2016; Tang et al., 2016) and increased anti-inflammatory cytokines, such as interleukin-4 (IL-4) and interleukin-10 (IL-10) (Jiang et al., 2017). The α7 nicotinic acetylcholine receptor (α7nAChR) is highly expressed in the central nervous system (CNS) and plays an important role in cholinergic anti-inflammatory pathway. Studies have showed that activating α7nAChR in microglia and macrophage inhibits the release of NO and TNF-α (Lutz et al., 2014; Baez-Pagan et al., 2015), suggesting that α7nAChR might be a target to regulate microglial phenotypes. Our previous study found that activating α7nAChR reduced cerebral ischemic injury (Wang et al., 2012). Therefore, we investigated the roles of microglial α7nAChR in the phenotypic conversion of microglia and the neuroprotective effects of EA pretreatment against ischemic stroke.

## Materials and Methods

### Animals

Male Sprague–Dawley (SD) rats (weighing 280–300 g) were obtained from the Experimental Animal Center of the Xi’an Jiaotong University (Xi’an, Shaanxi Province, China) and were maintained under a 12-h light–dark cycle, a temperature of 21 ± 2°C, and humidity of 60–70% for at least 1 week before any drug treatment or surgery was conducted. All animal experiments were approved by the Ethics Committee for Animal Experimental Center of the Xi’an Jiaotong University and were conducted according to the Chinese National Institutes of Health Guide for the Care and Use of Laboratory Animals^1^.

### Culture of Cells and Co-culture of Neuron–Microglia

Culture of cells and co-culture of neuron–microglia were performed as previously described (Zhang Z. et al., 2018). Briefly, mixed primary glial cultures were isolated from the cerebral cortex of 1-day-old SD pups. After the meninges and hippocampus were removed, the cortical tissues were subjected to enzymatic digestion and mechanical isolation. Then, the mixed cells were passed through a 70-μm nylon mesh cell strainer and were seeded into the poly-L-lysine-coated cell culture flask in Dulbecco’s modified Eagle’s medium (DMEM) containing 10% fetal bovine serum (FBS) and 1% antibiotics (penicillin and streptomycin). Cells were maintained in a humidified incubator at 37°C enriched with 5% CO_2_ and fed every 3–5 days with fresh medium. After 14 days, microglia were harvested from the mixed glial cultures by mild shaking at 220 r/min for 1 h and were seeded into cell culture containers. The purity of the microglia was confirmed by Iba1 staining (Supplementary Figure S1).

Primary neurons were isolated from the cerebral cortices of E17–E19 rat embryos and were seeded into the poly-L-lysine-coated cell culture plates. Then, cells were cultured in Neurobasal medium (Gibco, Invitrogen Corp., Carlsbad, CA, United States) containing 2% B27, 1% glutamine, and 1% penicillin/streptomycin (Sigma–Aldrich, St. Louis, MO, United States). Cells were kept in a humidified incubator at 37°C enriched with 5% CO_2_.

For indirect co-culture of neuron–microglia, the primary microglia (microglia:neurons = 1:2) were added to 0.4-μm pore sized Transwell inserts (Costar, United States) 2 days after the neurons were seeded into the 24-well plates. After the primary microglia and neurons were co-cultured for 2 days, the microglia and neurons were separated to receive drugs or oxygen-glucose deprivation (OGD) treatment. Then, the neurons and microglia were co-cultured again for 24 h.

### Groups and Drug Treatment

*In vivo*, first, to observe the neuroprotection of EA pretreatment and whether EA pretreatment induced the phenotypic conversion of microglia from M1 to M2 after ischemia–reperfusion injury, rats were randomly divided into three groups: sham, middle cerebral artery occlusion (MCAO), and EA + MCAO. The rats in the EA + MCAO group received EA for five consecutive days and were then subjected to MCAO for 90 min 1 day after the last EA pretreatment. The infarction volume and neurological scores were assessed 72 h after reperfusion. The expressions of α7nAChR; M1 microglia markers iNOS, IL-1β, and cluster of differentiation 86 (CD86); and M2 microglia markers arginase-1 (Arg-1), transforming growth factor-β1 (TGF-β1), and cluster of differentiation 206 (CD206) in the ischemic penumbra were analyzed. The pro-inflammatory cytokine TNF-α and anti-inflammatory cytokine IL-10 in the ischemic penumbra were analyzed. Second, to determine whether α7nAChR was associated with the phenotypic conversion of microglia and the neuroprotection of EA pretreatment after ischemia–reperfusion injury, rats were randomly divided into the following groups: sham, MCAO, *N*-(3*R*)-1-azabicyclo[2.2.2]oct-3-yl-furo[2,3-*c*]pyridine-5-carboxamide (PHA-543,613) + MCAO, vehicle + MCAO (saline), EA + MCAO, and sham, MCAO, EA + MCAO, α-bungarotoxin (α-BGT) + EA + MCAO, and vehicle + EA + MCAO (saline). Rats in the PHA-543,613 + MCAO group received intraperitoneal injection of PHA-543,613 (1.0 mg/kg, Tocris Bioscience, Bristol, United Kingdom) once a day for five consecutive days (Wang et al., 2012) and were then subjected to MCAO 24 h after the last injection. Rats in the α-BGT + EA + MCAO group received intracerebroventricular injection of α-BGT (0.5 μg/kg, Tocris Bioscience, Bristol, United Kingdom) for 30 min before the onset of EA for five consecutive days (Wang et al., 2012) and were then subjected to MCAO 24 h after the last EA treatment. The expressions of M1 microglia markers (iNOS, IL-1β, and CD86), M2 microglia markers (Arg-1, TGF-β1, and CD206), pro-inflammatory cytokine TNF-α, and anti-inflammatory cytokine IL-10 in the ischemic penumbra were accordingly analyzed. The infarction volume and neurological scores were assessed 72 h after reperfusion.

*In vitro*, to further confirm whether the α7nAChR in microglia was associated with the conversion of microglial phenotypes, α7nAChR agonist PHA-543,613 (100 μM) and antagonist α-BGT (10 nM) were used. Primary cultured microglia were randomly divided into five groups: control, OGD, PHA-543,613 + OGD, α-BGT + PHA-543,613 + OGD, and α-BGT + OGD. The PHA-543,613 (100 μM) was added into the cultured medium of microglia 24 h before OGD treatment, and the α7nAChR antagonist α-BGT (10 nM) was added to cells 30 min prior to PHA-543,613 to block the α7nAChR (Macpherson et al., 2014; Kimura et al., 2019). The M1 microglia markers (IL-1β and CD86) and M2 microglia markers (Arg-1 and CD206) were detected by western blot analysis and immunofluorescence staining. In addition, a neuron–microglia co-culture system was established to investigate the neuroprotection of α7nAChR in microglia. After the primary microglia and neurons were co-cultured for 2 days, the microglia were subjected to PHA-543,613 or α-BGT separately, and then the primary microglia and neurons were exposed to OGD for 4 and 1 h, respectively (Zhang Z. et al., 2018). The pro-inflammatory cytokines (TNF-α and IL-6) and anti-inflammatory cytokines (IL-4 and IL-10) in the supernatants of cultured microglia were detected by enzyme-linked immunosorbent assay (ELISA). The cell viability and lactate dehydrogenase (LDH) release of neurons were detected 24 h after the reintroduction of oxygen and glucose.

All drugs were administered as previously described (Wang et al., 2012; Macpherson et al., 2014; Kimura et al., 2019). All experiments followed the principles of randomization and double blindness.

### Intracerebral-Ventricular Injection

The rats were anesthetized with isoflurane and fixed on a stereotaxic apparatus (Narishige, Tokyo, Japan). A scalp incision was made, and the bregma was exposed. The stereotaxic apparatus was located at bregma and punched in the position of 1.5 mm lateral and 1.0 mm posterior to bregma at the left hemisphere. A stainless steel 26-gauge cannula (C315G; Plastics One Inc., Roanoke, VA, United States) was slowly introduced through the hole into the left lateral ventricle (3.8 mm below the cerebral dura mater). The cannula was fixed by dental cement, and four stainless steel screws were secured to the skull and occluded. The rats were allowed to recover from the operation 3 days before the next treatment.

### Electroacupuncture Pretreatment

Electroacupuncture was conducted as previously described (Li et al., 2012). Briefly, the rats were anesthetized, fixed on the animal thermostatic operating table, and maintained at 37.0 ± 0.5°C. The acupoint “Baihui (GV 20),” which is located at the intersection of the sagittal midline and the line linking the two ears, was stimulated using the Hwato Electronic Acupuncture Treatment Instrument (model no. SDZ-V, Suzhou Medical Appliances Co., Ltd., Suzhou, China) at an intensity of 1 mA and dense-disperse frequency of 2/15 Hz for 30 min per day for 5 days (Li et al., 2012).

### Middle Cerebral Artery Occlusion

The focal cerebral ischemia model of rats was induced by MCAO as described previously (Wang et al., 2009). Briefly, the rats were anesthetized with isoflurane. The right internal carotid artery was separated and occluded using an intraluminal filament technique. Regional cerebral blood flow (rCBF) was monitored using a transcranial Doppler ultrasound flowmeter (PeriFlux System 5000; PERIMED, Stockholm, Sweden). The body temperature of the rats was maintained at 37.0–37.5°C. The rats were available if the rCBF showed a sharp drop to 20 and were recovered up to more than 80% of baseline (pre-ischemia) level. There were no significant differences in physiological parameters during EA pretreatment (at the onset of EA, 15 min after EA, and at the end of EA) or surgery (at the onset of ischemia, 60 min after the onset of ischemia, and 30 min after reperfusion) according to our previous study (Wang et al., 2012).

### Neurobehavioral Evaluation

The neurological score was assessed 72 h after reperfusion using an 18-point neurobehavioral scoring system, which contained spontaneous activity, limb symmetry, forepaw outstretching, climbing, body proprioception, and response to vibrissae touch by another experimenter who was blind to the experimental groups as previously described (Garcia et al., 1995). The details of the score criterion are provided in Supplementary Table S1.

### Infarct Measurement

The infarct measurement was assessed by 2,3,5-triphenyltetrazolium chloride (TTC; Sigma–Aldrich, St. Louis, MO, United States) staining. The brain sections were stained for 15 min at 37°C, followed by overnight immersion in 4% paraformaldehyde. The stained sections were photographed, and the unstained areas were measured as the infarcted volume using an image analysis software (Adobe Photoshop 8.0 CS, Adobe Systems, San Jose, CA, United States) by an experimenter who was blind to the experimental groups. Infarct volume was quantified as follows: relative infarct volume = (contralateral area − ipsilateral non-infarct area)/contralateral area.

### Oxygen-Glucose Deprivation

The cells were switched to a serum- and glucose-free medium and were then transferred into a humidified hypoxic chamber, which contained mixed gas (5% CO_2_ and 95% N_2_) for 15 min at room temperature. The chamber was then sealed and placed in a container at 37°C. The OGD was carried out for 4 h (microglia) or for 1 h (neurons). Following the OGD, cells were incubated with normal medium for an additional 24 h after the reintroduction of oxygen and glucose.

### Assessment of Neuron Cell Viability

The survival of neuron cells was evaluated by cell counting kit-8 (CCK-8) (Seven Sea Biotech, China) 24 h after OGD. The procedures were strictly conducted according to the manufacturer’s instructions. Briefly, after the CCK-8 solution was added to each well of the 24-well plates, neurons were incubated for 4 h at 37°C. Eventually, the absorbance was measured at 450 nm using a microplate reader (Infinite M200; TECAN, Männedorf, Switzerland).

### Lactate Dehydrogenase Release Assay

The LDH-Cytotoxicity Colorimetric Assay Kit II (#K313-500; BioVision Inc., Milpitas, CA, United States) was used to detect cell injury 24 h after OGD. The LDH reaction mixture was mixed according to the manufacturer’s instructions and added to a 96-well plate (100 μl per well). Then, 10 μl of cell-free supernatant of the samples was incubated with this reaction mixture for 30 min at 37°C. Finally, the absorbance was measured at 450 nm using a microplate reader (Infinite M200; TECAN, Männedorf, Switzerland).

### Analysis of Inflammatory Factors

Enzyme-linked immunosorbent assay (R&D Systems, Minneapolis, MN, United States) was used to measure the levels of TNF-α and IL-10 in the ischemic penumbra, and also the levels of TNF-α, IL-4, IL-6, and IL-10 in the supernatants of cultured microglia according to the manufacturer’s instructions.

### Immunofluorescence Staining

Immunofluorescence staining was performed on frozen coronal sections of rat brains or on primary cultured microglia plated on cover slips. The rat brains were fixed with 4% paraformaldehyde. After fixation and concentration gradient dehydration, the brains were cut into 12-μm-thick sections using a Leica CM1900 frozen slicer. The cells were fixed with 4% paraformaldehyde for 30 min. The brain sections and cell cover slips were washed three times with phosphate-buffered saline (PBS) and then incubated overnight at 4°C in a humidified atmosphere with primary antibodies. The following primary antibodies were used: rabbit anti-iNOS antibody (1:100; Abcam, Cambridge, United Kingdom), rabbit anti-liver arginase antibody (1:100; Abcam, Cambridge, United Kingdom), rabbit anti-IL-1β antibody (1:50; Santa Cruz Biotechnology, Dallas, TX, United States), rabbit anti-α7nAChR antibody (1:50; Santa Cruz Biotechnology, Dallas, TX, United States), and goat anti-Iba1 antibody (1:50; Santa Cruz Biotechnology, Dallas, TX, United States) were used. The Alexa Fluor 488-labeled donkey anti-goat secondary antibody (1:200; Santa Cruz Biotechnology, Dallas, TX, United States), Alexa Fluor 594-labeled goat anti-rabbit secondary antibody (1:200; Santa Cruz Biotechnology, Dallas, TX, United States), and Alexa Fluor 488-labeled goat anti-rabbit secondary antibody (1:200; Santa Cruz Biotechnology, Dallas, TX, United States) were incubated for 2 h at room temperature in the dark. 4′,6-Diamidino-2-phenylindole (DAPI) (ZLI-9557, Zsbio) was used to stain nuclei. The sections were mounted with 50% glycerol. Finally, the sections were viewed and photographed using an Olympus BX51 (Japan) fluorescence microscope.

### Western Blot Analysis

To determine the expressions of α7nAChR, iNOS, IL-1β, CD86, Arg-1, TGF-β1, and CD206 in the ischemic penumbra of rats or in the microglia, proteins were extracted and western blot analysis was performed. Briefly, the extracted proteins were separated by 10% sodium dodecyl sulfate–polyacrylamide gel electrophoresis (SDS-PAGE) and electrically transferred to polyvinylidene difluoride (PVDF) membranes. Then, the membranes were blocked in TBST containing 5% non-fat dry milk for 1 h at room temperature and were successively incubated with the dilute primary and secondary antibodies. The following primary antibodies were used: anti-α7nAChR rabbit antibody (1:50; Santa Cruz Biotechnology, Dallas, TX, United States), anti-iNOS rabbit antibody (1:500; Abcam, Cambridge, United Kingdom), anti-IL-1β rabbit antibody (1:200; Santa Cruz Biotechnology, Dallas, TX, United States), anti-CD86 rabbit antibody (1:500; Proteintech, United States), anti-liver arginase rabbit antibody (1:1,000; Abcam, Cambridge, United Kingdom), an anti-TGF-β1 rabbit antibody (1:1,000; GeneTex, Irvine, CA, United States), anti-CD206 rabbit antibody (1:500; Proteintech, United States), and an anti-tubulin rabbit antibody (1:1,000; Beijing ComWin Biotech Co., Ltd, Beijing, China). In addition, horseradish peroxidase-conjugated goat anti-rabbit secondary antibodies (1:10,000; Beijing ComWin Biotech Co., Ltd, Beijing, China) were used. Protein bands were visualized using the LI-COR Odyssey System (LI-COR Biotechnology, Lincoln, NE, United States). The relative changes in protein expression were expressed as the ratio of the integrated optical density (OD) of the target protein band to that of β-tubulin.

### Statistical Analysis

GraphPad Prism 7.0 software was used for performing statistical analysis. Data were collected by two independent and blind investigators. Comparisons among multiple groups were undertaken using one-way ANOVA followed by Tukey’s *post hoc* test. Neurological scores were presented as medians and were analyzed using two-tailed Mann–Whitney *U*-tests, and the other values were presented as the mean ± SD. *p*-Value < 0.05 was considered statistically significant.

## Results

### Electroacupuncture Pretreatment Ameliorated Cerebral Ischemia Injury and Upregulated Microglial α7 Nicotinic Acetylcholine Receptor Expression in Ischemic Penumbra After Stroke

**FIGURE 1:**
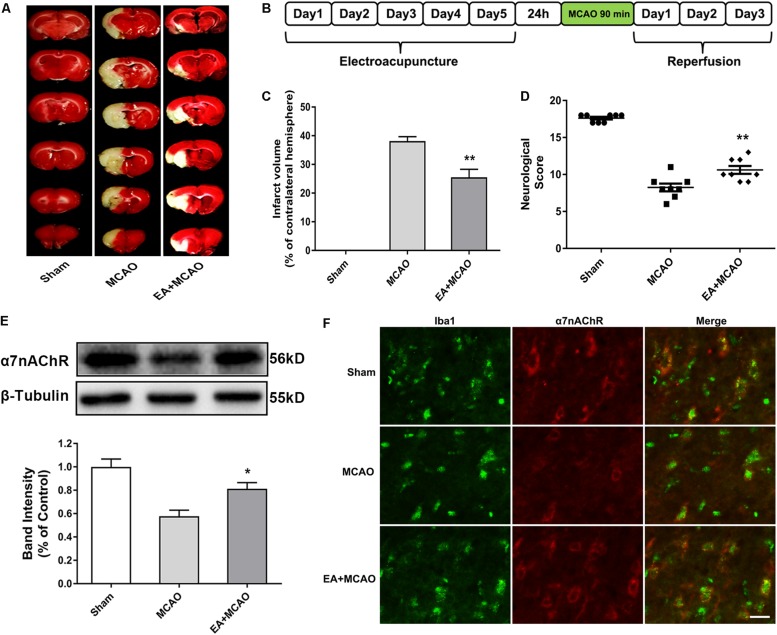
Electroacupuncture (EA) pretreatment ameliorated cerebral ischemia injury and upregulated α7 nicotinic acetylcholine receptor (α7nAChR) expression in ischemic penumbra after stroke. **(A)** 2,3,5-Triphenyltetrazolium chloride (TTC) staining was used to measure infarct volume in coronal brain sections from sham, middle cerebral artery occlusion (MCAO), and MCAO + EA-treated rats at 72 h after reperfusion. **(B)** Schematic diagram of EA pretreatment. EA stimulation parameters: density-sparse wave of 2/15 Hz, current intensity of 1 mA, and 30 min/day for five consecutive days. **(C)** The percentages of infarct volume. The data were expressed as the mean ± *SD* and were analyzed by one-way ANOVA with Tukey’s *post hoc* test. *n* = 8. ^∗∗^*p* < 0.01 compared with the MCAO group. **(D)** Neurological deficit scores were evaluated 72 h after reperfusion. The data were expressed as the median and were analyzed by the Mann–Whitney *U*-test. *n* = 8. ^∗∗^*p* < 0.01 compared with the MCAO group. **(E)** Western blot analysis of the expression level of α7nAChR protein in the ischemic penumbra 72 h after reperfusion. The data were expressed as the mean ± *SD* and were analyzed by one-way ANOVA with Tukey’s *post hoc* test. *n* = 5. ^∗^*p* < 0.05 compared with the MCAO group. **(F)** Representative immunofluorescence images showing the expression of α7nAChR in microglia in the ischemic penumbra after stroke. Microglial cells were labeled by Iba1 (microglia marker, green). *n* = 5. Scale bars = 20 μm.

The influence of EA pretreatment on cerebral ischemia injury and α7nAChR expression in the ischemic penumbra was evaluated 72 h after reperfusion (Figure 1B). As shown in Figure 1, EA pretreatment, compared with the MCAO group, significantly ameliorated the infarction volume (Figures 1A,C, ^∗∗^*p* < 0.01) and increased the neurological scores after stroke, which mean that the neurological deficits had been improved (Figure 1D, ^∗∗^*p* < 0.01). Moreover, the protein expression of α7nAChR in the ischemic penumbra was significantly increased in the EA group compared with the MCAO group (Figure 1E, ^∗^*p* < 0.05). In addition, we detected the expression of α7nAChR in microglia by immunofluorescent double labeling of α7nAChR and Iba1 (microglial marker). It showed that EA pretreatment, compared with the MCAO group, upregulated microglial α7nAChR expression in the ischemic penumbra (Figure 1F). These results indicated that EA pretreatment exerted neuroprotective effects and reversed the effects of MCAO on the expression of α7nAChR in the ischemic penumbra of rats subjected to ischemia injury.

### Electroacupuncture Pretreatment Induced the Phenotypic Conversion of Microglia From M1 to M2 and Relieved Inflammatory Response in the Ischemic Penumbra After Stroke

**FIGURE 2:**
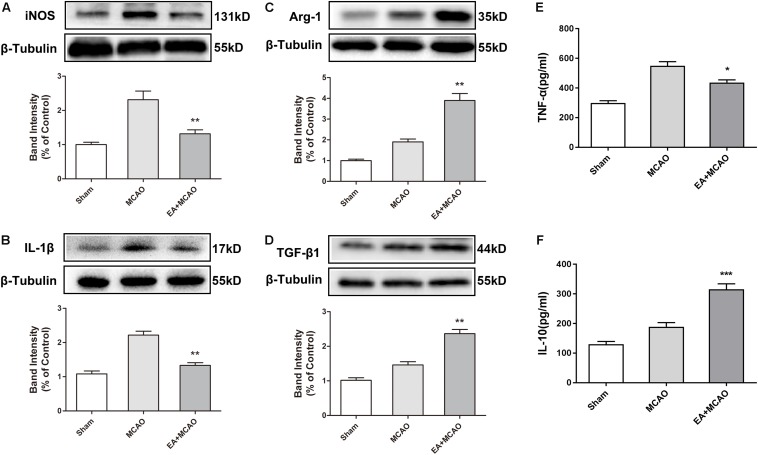
Electroacupuncture (EA) pretreatment induced the phenotypic conversion of microglia from M1 to M2 and relieved inflammatory response in the ischemic penumbra after stroke. **(A–D)** Western blot analysis of the expression of M1 microglia markers nitric oxide synthase (iNOS) and interleukin-1β (IL-1β) as well as M2 microglia markers arginase-1 (Arg-1) and transforming growth factor-β1 (TGF-β1) in the ischemic penumbra 72 h after reperfusion. The data were expressed as the mean ± *SD* and were analyzed by one-way ANOVA with Tukey’s *post hoc* test. *n* = 5. ^∗∗^*p* < 0.01 compared with the MCAO group. **(E,F)** The levels of pro-inflammatory cytokine tumor necrosis factor-α (TNF-α) and anti-inflammatory cytokine interleukin-10 (IL-10). The data were expressed as the mean ± *SD* and were analyzed by one-way ANOVA with Tukey’s *post hoc* test. *n* = 5. ^∗^*p* < 0.05, ^∗∗∗^*p* < 0.001 compared with the MCAO group.

The time point of 72 h after ischemia–reperfusion was the key time point for microglial transformation from M1 to M2 (Zhai et al., 2017); thus, this specific time point was selected for subsequent experiments. At 72 h after ischemia–reperfusion, the expression of M1 microglia markers iNOS and IL-1β in the ischemic penumbra were significantly decreased in the EA + MCAO group compared with the MCAO group (Figures 2A,B, ^∗∗^*p* < 0.01), whereas the expressions of M2 microglia markers Arg-1 and TGF-β1 were remarkably increased (Figures 2C,D, ^∗∗^*p* < 0.01), which indicated that EA pretreatment induced the phenotypic conversion of microglia from M1 to M2. The pro-inflammatory cytokine TNF-α was significantly decreased and anti-inflammatory cytokine IL-10 was notably increased after EA pretreatment in the ischemic penumbra as detected by ELISA (Figures 2E,F, ^∗^*p* < 0.05, ^∗∗∗^*p* < 0.001).

### The α7 Nicotinic Acetylcholine Receptor Was Involved in the Conversion of the Microglial Phenotype After Oxygen-Glucose Deprivation *in vitro*

**FIGURE 3:**
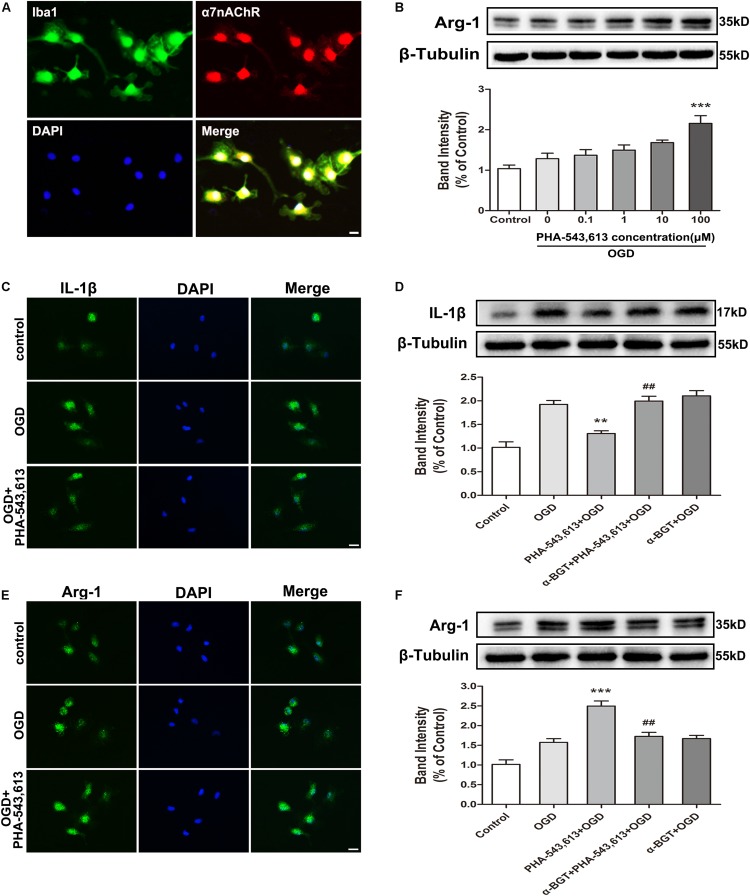
α7 nicotinic acetylcholine receptor (α7nAChR) was involved in the modulation of the microglial phenotype after oxygen-glucose deprivation (OGD) *in vitro*. **(A)** Representative immunofluorescence images showing the expression of α7nAChR in primary microglia. Scale bar = 5 μm. **(B)** Western blot analysis of the expression levels of arginase-1 (Arg-1) protein in primary microglia exposed to different doses of PHA-543,613 24 h after the reintroduction of oxygen and glucose. The α7nAChR agonist PHA-543,613 was administrated 24 h before OGD. The data were expressed as the mean ± *SD* and were analyzed by one-way ANOVA with Tukey’s *post hoc* test. The data were pooled from five independent experiments. ^∗∗∗^*p* < 0.001 compared with the OGD group without drug treatment. **(C)** Immunofluorescence staining of M1 microglia marker IL-1β in primary microglia 24 h after the reintroduction of oxygen and glucose. The α7nAChR agonist PHA-543,613 (100 μM) was administrated 24 h before OGD. Scale bar = 5 μm. **(D)** Western blot analysis of the protein expression of IL-1β in primary microglia 24 h after the reintroduction of oxygen and glucose. The α7nAChR agonist PHA-543,613 (100 μM) was administrated 24 h before OGD. The α7nAChR antagonist α-BGT (10 nM) was administrated 30 min prior to PHA-543,613 to block the function of α7nAChR. The data were expressed as the mean ± *SD* and were analyzed by one-way ANOVA with Tukey’s *post hoc* test. The data were pooled from five independent experiments. ^∗∗^*p* < 0.01 compared with the OGD group, ^##^*p* < 0.01 compared with the PHA-543,613 + OGD group. **(E)** Immunofluorescence staining of M2 microglia marker Arg-1 in primary microglia 24 h after the reintroduction of oxygen and glucose. Scale bar = 5 μm. **(F)** Western blot analysis of the protein expression of Arg-1 in primary microglia 24 h after the reintroduction of oxygen and glucose. The data were expressed as the mean ± *SD* and were analyzed by one-way ANOVA with Tukey’s *post hoc* test. The data were pooled from five independent experiments. ^∗∗∗^*p* < 0.001 compared with the OGD group, ^##^*p* < 0.01 compared with the PHA-543,613 + OGD group.

We used primary microglia to further investigate whether the microglial phenotypic conversion was caused by the activation of α7nAChR in microglia. The purity of cultured primary microglia was confirmed by Iba1 staining (Supplementary Figure S1). The α7nAChR was expressed in the microglia (Figure 3A). Compared with the OGD group without drug treatment, pretreating microglia with 100 μM of α7nAChR agonist PHA-543,613 24 h before OGD treatment induced the phenotypic conversion of microglia from M1 to M2 as assessed by the expression of Arg-1 in microglia exposed to OGD injury (Figure 3B, ^∗∗∗^*p* < 0.001); thus, the concentration of PHA-543,613 was chosen for the following experiments. PHA-543,613 pretreatment significantly attenuated M1 microglia markers IL-1β and CD86 protein expressions (Figures 3C,D and Supplementary Figure S2C, ^∗∗^*p* < 0.01) and increased M2 microglia markers Arg-1 and CD206 expressions (Figures 3E,F and Supplementary Figure S2C, ^∗∗∗^*p* < 0.01) in the microglia exposed to OGD. When the function of α7nAChR was blocked by adding its antagonist α-BGT to cells 30 min prior to PHA-543,613, PHA-543,613 pretreatment lost its influence on the expression of M1 and M2 microglia markers (Figures 3C–F and Supplementary Figure S2C, PHA-543,613 + OGD vs. α-BGT + PHA-543,613 + OGD). No significant differences were found between the OGD group and the α-BGT + PHA-543,613 + OGD group. These results suggested that microglial α7nAChR contributed to the phenotypic conversion of microglia.

### Activating Microglial α7 Nicotinic Acetylcholine Receptor Relieved Inflammatory Response and Induced Neuroprotection Against Oxygen-Glucose Deprivation Injury *in vitro*

**FIGURE 4:**
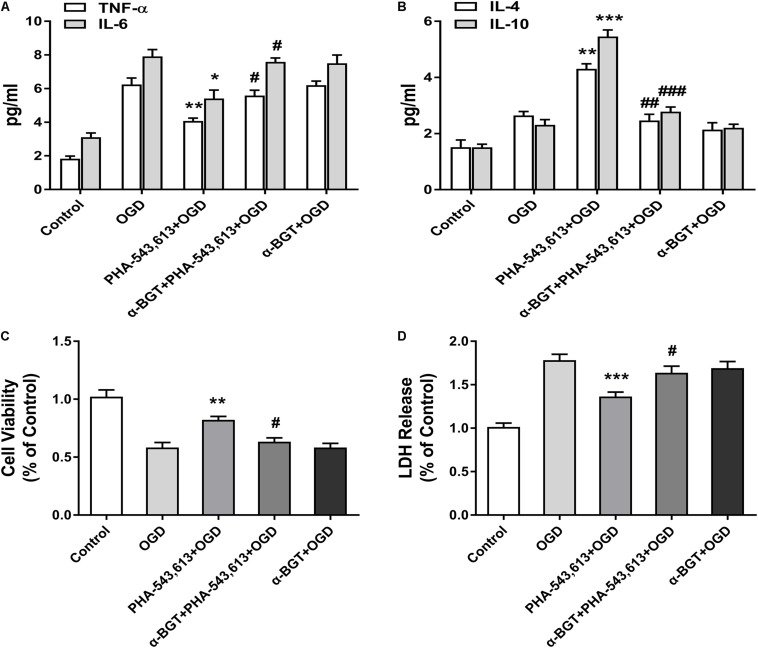
Activating α7 nicotinic acetylcholine receptor (α7nAChR) in microglia relieved inflammatory response and induced neuroprotection against oxygen-glucose deprivation (OGD) injury *in vitro*. **(A)** The levels of the pro-inflammatory cytokines tumor necrosis factor-α (TNF-α) and interleukin-6 (IL-6) in the supernatants of primary microglia were detected by ELISA 24 h after the reintroduction of oxygen and glucose. The data were expressed as the mean ± *SD* and were analyzed by one-way ANOVA with Tukey’s *post hoc* test. The data were pooled from five independent experiments. ^∗^*p* < 0.05, ^∗∗^*p* < 0.01 compared with the OGD group, ^#^*p* < 0.05 compared with the PHA-543,613 + OGD group. **(B)** The levels of the anti-inflammatory cytokines IL-4 and IL-10 in the supernatants of primary microglia were detected by ELISA 24 h after the reintroduction of oxygen and glucose. The data were expressed as the mean ± *SD* and were analyzed by one-way ANOVA with Tukey’s *post hoc* test. The data were pooled from five independent experiments. ^∗∗^*p* < 0.01, ^∗∗∗^*p* < 0.001 compared with the OGD group, ^##^*p* < 0.01, ^###^*p* < 0.001 compared with the PHA-543,613 + OGD group. **(C)** The cell viability of neurons co-cultured with microglia was detected by cell counting kit-8 (CCK-8) 24 h after the reintroduction of oxygen and glucose. The data were expressed as the mean ± *SD* and were analyzed by one-way ANOVA with Tukey’s *post hoc* test. The data were pooled from five independent experiments. ^∗∗^*p* < 0.01 compared with the OGD group, ^#^*p* < 0.05 compared with the PHA-543,613 + OGD group. **(D)** The injury of neurons co-cultured with microglia was detected by lactate dehydrogenase (LDH) release assay 24 h after the reintroduction of oxygen and glucose. The data were expressed as the mean ± SD and were analyzed by one-way ANOVA with Tukey’s *post hoc* test. The data were pooled from five independent experiments. ^∗∗∗^*p* < 0.001 compared with the OGD group, ^#^*p* < 0.05 compared with the PHA-543,613 + OGD group.

The levels of pro-inflammatory cytokines TNF-α and IL-6 and the levels of anti-inflammatory cytokines IL-10 and IL-4 in the supernatants of primary microglia were detected 24 h after the reintroduction of oxygen and glucose. The levels of TNF-α and IL-6 were significantly decreased whereas the levels of IL-4 and IL-10 were remarkably increased in the PHA-543,613 + OGD group when compared with the OGD group (Figures 4A,B, ^∗^*p* < 0.05, ^∗∗^*p* < 0.01, ^∗∗∗^*p* < 0.001). After the function of α7nAChR was blocked by its antagonist α-BGT, the PHA-543,613 pretreatment lost its influence on the expressions of TNF-α, IL-6, IL-4, and IL-10 (Figures 4A,B; PHA-543,613 + OGD vs. α-BGT + PHA-543,613 + OGD). To further observe the neuroprotection of microglial α7nAChR, neurons and microglia were co-cultured. During the drug treatments and OGD, neurons and microglia were fully separated to avoid activating α7nAChR in neurons owing to the leakage of drugs through the 0.4-μm pore-sized membrane of the Transwell inserts. According to the CCK-8 cell viability assay, activation of α7nAChR in microglia significantly improved the viability of neurons compared with that in the OGD group (Figure 4C, ^∗∗^*p* < 0.01). According to the LDH release assay, the levels of LDH release in the PHA-543,613 + OGD group were markedly lower than those in the OGD group (Figure 4D, ^∗∗∗^*p* < 0.001). After the function of α7nAChR was blocked by its antagonist α-BGT, the PHA-543,613 pretreatment lost its influence on the viability and injury of neurons exposed to OGD (Figures 4C,D; PHA-543,613 + OGD vs. α-BGT + PHA-543,613 + OGD). No significant differences were observed between the OGD group and the α-BGT + PHA-543,613 + OGD group. These results indicated that activation of α7nAChR relieved inflammatory response and protected neurons from OGD injury.

### Electroacupuncture Pretreatment Induced the Phenotypic Conversion of Microglia From M1 to M2 via α7 Nicotinic Acetylcholine Receptor After Stroke

**FIGURE 5:**
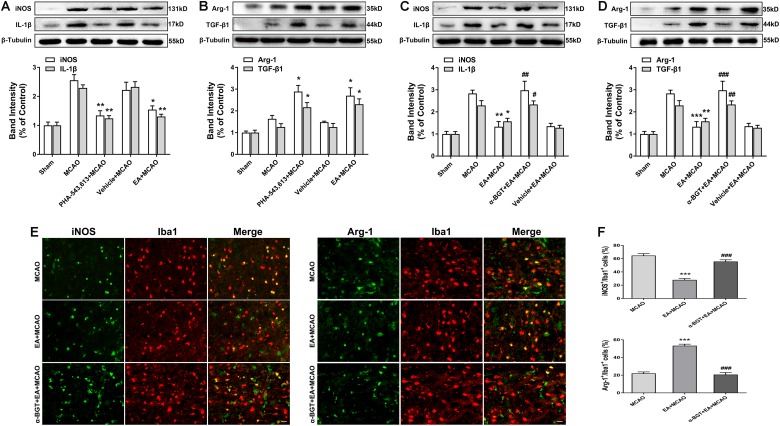
Electroacupuncture (EA) pretreatment induced the phenotypic conversion of microglia from M1 to M2 via α7 nicotinic acetylcholine receptor (α7nAChR) after stroke. **(A)** Western blot analysis of the expression of M1 microglia markers nitric oxide synthase (iNOS) and interleukin-1β (IL-1β) in the ischemic penumbra 72 h after reperfusion. The data were expressed as the mean ± *SD* and were analyzed by one-way ANOVA with Tukey’s *post hoc* test. *n* = 5. ^∗^*p* < 0.05, ^∗∗^*p* < 0.01 compared with the middle cerebral artery occlusion (MCAO) group. **(B)** Western blot analysis of the expression of M2 microglia markers arginase-1 (Arg-1) and transforming growth factor-β1 (TGF-β1) in the ischemic penumbra 72 h after reperfusion. The data were expressed as the mean ± *SD* and were analyzed by one-way ANOVA with Tukey’s *post hoc* test. *n* = 5. ^∗^*p* < 0.05 compared with the MCAO group. **(C)** Western blot analysis of the expression of M1 microglia markers iNOS and IL-1β in the ischemic penumbra 72 h after reperfusion. The data were expressed as the mean ± *SD* and were analyzed by one-way ANOVA with Tukey’s *post hoc* test. *n* = 5. ^∗^*p* < 0.05, ^∗∗^*p* < 0.01 compared with the MCAO group, ^#^*p* < 0.05, ^##^*p* < 0.01 compared with the EA + MCAO group. **(D)** Western blot analysis of the expression of M2 microglia markers Arg-1 and TGF-β1 in the ischemic penumbra 72 h after reperfusion. The data were expressed as the mean ± *SD* and were analyzed by one-way ANOVA with Tukey’s *post hoc* test. *n* = 5. ^∗∗^*p* < 0.01, ^∗∗∗^*p* < 0.001 compared with the MCAO group, ^##^*p* < 0.01, ^###^*p* < 0.001 compared with the EA + MCAO group. **(E)** Representative immunofluorescence images showing the expression of iNOS and Arg-1 in microglial cells. *n* = 5. Scale bar = 20 μm. **(F)** The percentage of iNOS^+^/Iba1^+^ and Arg-1^+^/Iba1^+^ cells. The data were expressed as the mean ± SD and were analyzed by one-way ANOVA with Tukey’s *post hoc* test. *n* = 5. ^∗∗∗^*p* < 0.001 compared with the MCAO group, ^###^*p* < 0.001 compared with the EA + MCAO group.

The α7nAChR agonist PHA-543,613 and antagonist α-BGT were used to detect whether α7nAChR was associated with the phenotypic conversion of microglia *in vivo*. As illustrated in Figure 5, 72 h after ischemia–reperfusion, the expressions of M1 microglia markers iNOS, IL-1β, and CD86 in the ischemic penumbra were significantly reduced in the PHA-543,613 + MCAO group compared with the MCAO group (Figure 5A and Supplementary Figure S2A, ^∗^*p* < 0.05, ^∗∗^*p* < 0.01), whereas the expressions of M2 microglia markers Arg-1, TGF-β1, and CD206 were notably increased (Figure 5B and Supplementary Figure S2A, ^∗^*p* < 0.05, ^∗∗∗^*p* < 0.001). The EA and PHA-543,613 pretreatment had the same influence on the expression of iNOS, IL-1β, CD86, Arg-1, TGF-β1, and CD206 (Figures 5A,B and Supplementary Figure S2A). The expressions of M1 microglia markers iNOS, IL-1β, and CD86 in the ischemic penumbra were markedly increased in the α-BGT + EA + MCAO group compared with the EA + MCAO group (Figure 5C and Supplementary Figure S2B, ^#^*p* < 0.05, ^##^*p* < 0.01, ^###^*p* < 0.001), whereas the expression of M2 microglia markers Arg-1, TGF-β1, and CD206 were significantly decreased (Figure 5D and Supplementary Figure S2B, ^ #^*^#^p* < 0.01, ^ #^*^##^p* < 0.001). The immunofluorescence assay revealed that the percentage of iNOS^+^/Iba1^+^ microglia in the ischemic penumbra of the EA + MCAO group was less than that in the MCAO group, and the percentage of Arg-1^+^/Iba1^+^ microglia in the ischemic penumbra of the EA + MCAO group was more than that in the MCAO group (Figures 5E,F, ^∗∗∗^*p* < 0.001). After the function of α7nAChR was blocked by its antagonist α-BGT, the EA pretreatment lost its influence on the percentage of iNOS^+^/Iba1^+^ microglia and Arg-1^+^/Iba1^+^ microglia in the ischemic penumbra (Figures 3E,F, α-BGT + EA + MCAO vs. EA + MCAO). No significant differences were observed between the MCAO group and the α-BGT + EA + MCAO group. These findings suggested that EA pretreatment induced the phenotypic conversion of microglia from M1 to M2 via α7nAChR.

### Electroacupuncture Pretreatment Relieved Inflammatory Response and Alleviated Cerebral Ischemia Injury via α7 Nicotinic Acetylcholine Receptor

**FIGURE 6:**
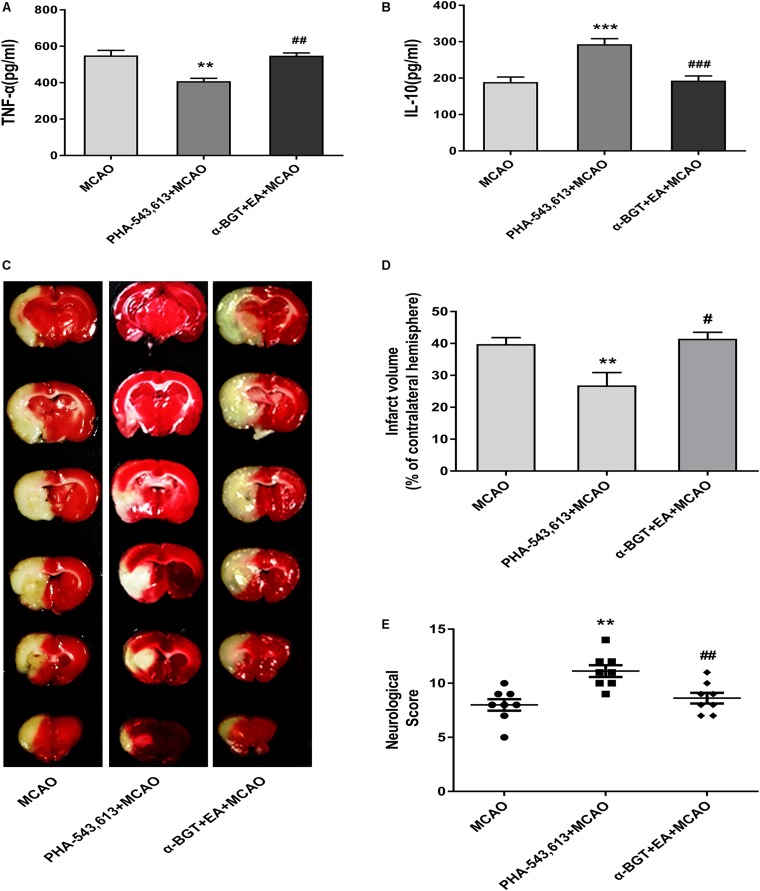
EA pretreatment relieved inflammatory response and alleviated cerebral ischemia injury via α7 nicotinic acetylcholine receptor (α7nAChR). **(A,B)** The level of the pro-inflammatory cytokine tumor necrosis factor-α (TNF-α) and anti-inflammatory cytokine interleukin-10 (IL-10) in the ischemic penumbra was detected 72 h after reperfusion by ELISA. The data were expressed as the mean ± *SD* and were analyzed by one-way ANOVA with Tukey’s *post hoc* test. *n* = 5. ^∗∗^*p* < 0.01, ^∗∗∗^*p* < 0.001 compared with the middle cerebral artery occlusion (MCAO) group, ^##^*p* < 0.01, ^###^*p* < 0.001 compared with the PHA-543,613 + MCAO group. **(C)** Representative photographs of brain slices showing the infarct volume assessed 72 h after reperfusion. **(D)** The percentages of infarct volume. The data were expressed as the mean ± *SD* and were analyzed by one-way ANOVA with Tukey’s *post hoc* test. *n* = 8. ^∗∗^*p* < 0.01 compared with the MCAO group, ^#^*p* < 0.05 compared with the PHA-543,613 + MCAO group. **(E)** Neurological deficit scores were evaluated 72 h after reperfusion. The data were expressed as the median and were analyzed by the Mann–Whitney *U*-test. *n* = 8. ^∗∗^*p* < 0.01 compared with the MCAO group, ^##^*p* < 0.01 compared with the PHA-543,613 + MCAO group.

The pro-inflammatory cytokine TNF-α and anti-inflammatory cytokine IL-10 in the ischemic penumbra 72 h after ischemia–reperfusion were measured by ELISA. The level of pro-inflammatory TNF-α was significantly decreased whereas the level of anti-inflammatory IL-10 was remarkably increased in the PHA-543,613 + MCAO group compared with the MCAO group (Figures 6A,B, ^∗∗^*p* < 0.01, ^∗∗∗^*p* < 0.001). The level of pro-inflammatory TNF-α was notably increased whereas the level of anti-inflammatory IL-10 was significantly decreased in the α-BGT + EA + MCAO group compared with the PHA-543,613 + MCAO group (Figures 6A,B, ^ ##^*p* < 0.01, ^###^*p* < 0.001).

The infarct volume was significantly decreased and the neurological deficit was dramatically relieved in the PHA-543,613 + MCAO group compared with the MCAO group, whereas the infarct volume was increased and the neurological deficit was aggravated in the α-BGT + EA + MCAO group compared with the PHA-543,613 + MCAO group (Figures 6C–E, ^∗∗^*p* < 0.01, ^#^*p* < 0.05, ^##^*p* < 0.01). No significant differences were observed between the MCAO group and the α-BGT + EA + MCAO group. These results further demonstrated that EA pretreatment displayed neuroprotective effect by regulating inflammatory response via α7nAChR.

## Discussion

In the present study, we investigated the roles of microglial α7nAChR in the neuroprotection of EA pretreatment against ischemic injury by *in vitro* and *in vivo* experiments. We found that EA pretreatment decreased the expression of M1 microglia markers (iNOS, IL-1β, and CD86) and pro-inflammatory cytokines (TNF-α and IL-6), whereas it increased the expression of M2 microglia markers (Arg-1, TGF-β1, and CD206) and anti-inflammatory cytokines (IL-4 and IL-10) in the ischemic penumbra and microglia by activating α7nAChR. EA pretreatment also significantly reduced the infarct volume and improved the neurological deficit. Activation of α7nAChR in microglia relieved the inflammatory response of primary microglia subjected to OGD and attenuated the injury of neurons co-cultured with microglia. Thus, although failing to completely cure stroke, EA pretreatment exerted neuroprotective effects against cerebral ischemic injury by promoting α7nAChR-mediated microglial phenotype conversion, suggesting a potential therapeutic target in the treatment of ischemic stroke.

Electroacupuncture confers strong neuroprotection against cerebral ischemic injury by inhibiting the inflammatory responses (Xiong et al., 2003; Wang et al., 2005, 2011; Hong et al., 2013; Zhu et al., 2013). A previous study has found that EA pretreatment suppresses the NF-κB signaling pathway by upregulating cylindromatosis to alleviate inflammatory injury after cerebral ischemia/reperfusion (Jiang et al., 2017). In addition, EA treatment inhibits microglia-mediated neuroinflammation through inactivation of p38 MAPK and MyD88, accompanied by the decrease of IL-1β, IL-6, and TNF-α after ischemic stroke (Liu et al., 2016). A randomized clinical trial provides evidences for the effectiveness of EA pretreatment on urinary leakage among women with stress urinary incontinence (Liu et al., 2017). In the current study, we demonstrated that EA pretreatment significantly decreased the expression of M1 microglia markers iNOS, IL-1β, and CD86, whereas it increased the expression of M2 microglia markers Arg-1, TGF-β1, and CD206 in the ischemic penumbra. Microglial cells are the pivotal mediators of neuroinflammation, performing functions by adopting different activation states, M1 phenotype with cytotoxic properties, and M2 phenotype with regeneration and repair (Colonna and Butovsky, 2017). The iNOS is transiently expressed during immune activation. Overproduction of NO by iNOS acts as a cytotoxic agent in pathological processes (Mukherjee et al., 2014). IL-1β is mainly produced by M1 microglia, expanding the microglia population in an autocrine manner and amplifying the production of inflammatory cytokines (Zhang C. J. et al., 2018). Arg-1 is expressed in the immune system and participates in a variety of inflammatory diseases by downregulation of nitric oxide synthesis, induction of fibrosis, and tissue regeneration (Munder, 2009). TGF-β1 has extensive immunomodulatory and anti-inflammatory properties and is abundantly produced in the ischemic penumbra by microglia (Islam et al., 2018). EA pretreatment transformed microglial phenotype from M1 to M2; thus, the expression of M1-related proteins decreased and M2-related proteins increased. Whether the inhibition of p38 MAPK-, MyD88-, and NF-κB-mediated signaling pathways is involved in the EA pretreatment-induced expression changes of iNOS, IL-1β, Arg-1, and TGF-β1 needs further investigation.

The α7nAChR is the pivotal receptor in the cholinergic anti-inflammatory pathway and is widely expressed in the CNS (Sharma and Vijayaraghavan, 2001; Gotti and Clementi, 2004; Shytle et al., 2004; Huston et al., 2006; De Jaco et al., 2017). Pharmacological stimulation of α7nAChR and stimulation of vagus nerve exert neuroprotective effects against cerebral ischemic injury (Kalappa et al., 2013; Parada et al., 2013; Jiang et al., 2014). Moreover, the α7nAChR signaling modulates the inflammatory phenotype of microglia in fetal brain, thereby providing a therapeutic approach for neuroinflammation *in utero* (Cortes et al., 2017). Consistent with these studies, we found that microglial α7nAChR played a key role in the neuroprotection of EA pretreatment against ischemic injury. In our study, activating α7nAChR in microglia promoted conversion of microglial phenotypes from M1 to M2 as assessed by the expression of M1 and M2 microglia markers, the pro-inflammatory cytokines (TNF-α and IL-6), and anti-inflammatory cytokines (IL-4 and IL-10) in the ischemic penumbra or in the supernatant of primary microglia. Furthermore, by establishing the microglia-neurons co-culture system, we found that activating α7nAChR in microglia increased neuronal cell viability and attenuated neuronal injury, which indicated that microglial α7nAChR exerted neuroprotection effects against OGD injury.

Microglial phenotypic transformation plays an important role in stroke, but the specific molecular mechanisms are not fully understood. A study has shown that cannabinoid receptors drive the acquisition of M2 polarization in microglia (Mecha et al., 2015). Inhibition of MyD88 signaling attenuates neuronal death in the hippocampus after status epilepticus in mice by skewing microglia/macrophage polarization (Liu et al., 2018). Our previous study found that activation or upregulation of triggering receptor expressed on myeloid cells 2 (TREM2) promoted the phenotypic conversion of microglia and thus decreased the number of apoptotic neurons in stroke mice (Zhai et al., 2017). Studies have also found that α7nAChR activation altered microglial phenotype by modulating intracellular iron load through α7nAChR-ferroportin signaling pathway, thus playing a neuroprotective role (Cortes et al., 2017). In addition, activation of the α7nAChR strongly inhibits the phosphorylation of p38 MAPK and upregulates phosphorylation of JAK2 and STAT3, thus transforming LPS-activated M1 microglia to the M2 phenotype (Zhang et al., 2017). Whether these pathways and signaling molecules are associated with the α7nAChR-mediated conversion of microglial phenotypes requires further clarification.

Our study includes some limitations. First, utilizing a rat model with a conditional knockout of the α7nAChR in microglia could better verify the effect of microglial α7nAChR. Second, as estrogen is an independent factor affecting the outcomes of stroke and the inflammatory signaling pathways, we failed to account for females. And long-term sensory or motor impairments detecting would be helpful to assess potential future therapies of EA pretreatment.

## Conclusion

We found that EA pretreatment alleviated cerebral ischemic injury through the α7nAChR-mediated phenotypic conversion of microglia, which might be a new mechanism for EA pretreatment-induced neuroprotection against cerebral ischemia.

## Data Availability Statement

The raw data supporting the conclusions of this article will be made available by the authors, without undue reservation, to any qualified researcher.

## Author Contributions

Study supervision and study design was performed by QW. The study was conducted by ZM and ZZ. Data collection and analysis were performed by FB, TJ, and CY. Writing and critical revision of the manuscript were performed by ZM, ZZ, and QW.

## Conflict of Interest

The authors declare that the research was conducted in the absence of any commercial or financial relationships that could be construed as a potential conflict of interest.

## Footnotes

1 https://grants.nih.gov/grants/olaw/Guide-for-the-Care-and-use-of-laboratory-animals.pdf
