# Supplementary material for: Electroacupuncture Pretreatment Alleviates Cerebral Ischemic Injury Through α7 Nicotinic Acetylcholine Receptor-Mediated Phenotypic Conversion of Microglia
Source: Front Cell Neurosci. 2019 Dec 6;13:537. doi: 10.3389/fncel.2019.00537 (PMC6908971; doi:10.3389/fncel.2019.00537)
Supplement: Supplementary file 1 [file Data_Sheet_1.pdf]

Supplementary Materials

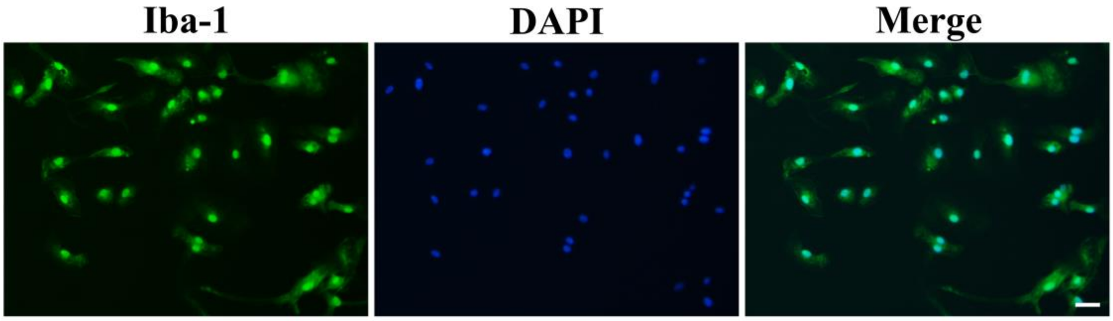

Fig. S1 The identification of primary microglial cells in culture.
The purity of the microglial cells in culture were more than 95%, as confirmed by staining with the microglia marker Iba-1 and DAPI. Scale bar=20 μm.

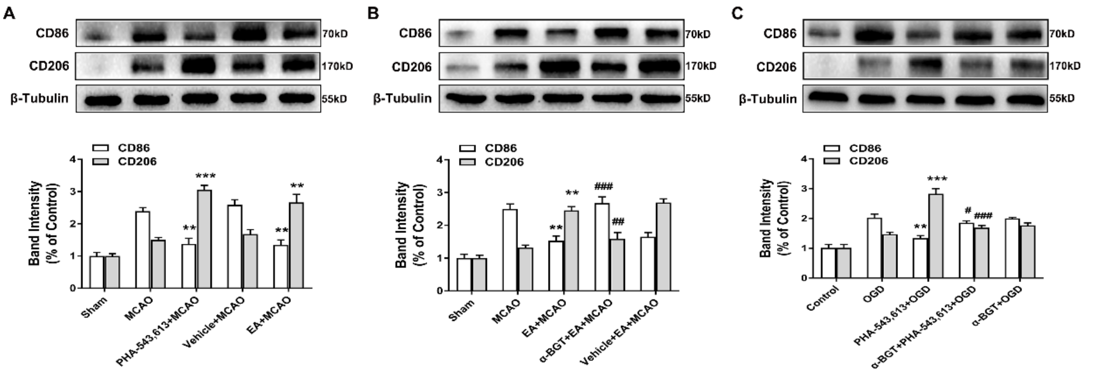

Fig. S2 The phenotypic conversion of microglia from M1 to M2 via α7nAChR after stroke or OGD.
(A) Western blot analysis of the expression of M1 microglia marker CD86 and M2 microglia marker CD206 in the ischemic penumbra 72 h after reperfusion. The data were expressed as the mean ± SD and were analyzed by one-way ANOVA with Tukey's post-hoc test. n = 5. $^{**}p < 0.01$, $^{***}p < 0.001$ compared with the MCAO group. (B) Western blot analysis of the expression of M1 microglia marker CD86 and M2 microglia marker CD206 in the ischemic penumbra 72 h after reperfusion. The data were expressed as the mean ± SD and were analyzed by one-way ANOVA with Tukey's post-hoc test. n = 5. $^{**}p < 0.01$ compared with the MCAO group. $^{\#\#}p < 0.01$, $^{\#\#\#}p < 0.001$ compared with the EA+MCAO group. (C) Western blot analysis of the protein expression of M1 microglia marker CD86 and M2 microglia marker CD206 in primary microglia 24 h after the reintroduction of oxygen and glucose. The α7nAChR agonist PHA-543,613 (100 μM) was administrated 24 h before OGD. The α7nAChR antagonist α-BGT (10 nM) was administrated 30 min prior to PHA-543,613 to block the function of α7nAChR. The data were expressed as the mean ± SD and were analyzed by one-way ANOVA with Tukey's post-hoc test. The data were pooled from five independent experiments. $^{**}p < 0.01$, $^{***}p < 0.001$ compared with the OGD group, $^{\#}p < 0.05$, $^{\#\#\#}p < 0.001$ compared with the PHA-543,613+OGD group.

Table. S1 Neurological Evaluation After Middle Cerebral Artery Occlusion

**Test**	**Score**			
	**0**	**1**	**2**	**3**
Spontaneous activity (in cage for 5 min)	No movement	Barely moves	Moves but does not approach at least three sides of cage	Moves and approaches at least three sides of cage
Symmetry of movements (four limbs)	Left side: no movement	Left side: slight movement	Left side: moves slowly	Both sides: move symmetrically
Symmetry of forelimbs (outstretching while held by tail)	Left side: no movement, no outreaching	Left side: slight movement to outreach	Left side: moves and outreaches less than right side	Symmetrical outreach
Climbing wall of wire cage	…	Fails to climb	Left side is weak	Normal climbing
Reaction to touch on either side of trunk	…	No response on left side	Weak response on left side	Symmetrical response
Response to vibrissae touch	…	No response on left side	Weak response on left side	Symmetrical response

Severe neurological dysfunction: 3-7 point; Moderate neurological dysfunction: 8-11 point; Mild neurological dysfunction: 12-18 point. Higher scores indicate better neurobehavioral outcomes in animals.

1. Spontaneous Activity

The animal was observed for 5 minutes in its normal environment (cage). The rat's activity was assessed by its ability to approach all four walls of the cage. Scores indicate the following: 3, rat moved around, explored the environment, and approached at least three walls of the cage; 2, slightly affected rat moved about in the

cage but did not approach all sides and hesitated to move, although it eventually reached at least one upper rim of the cage; 1, severely affected rat did not rise up at all and barely moved in the cage; and 0, rat did not move at all.

2. Symmetry in the Movement of Four Limbs

The rat was held in the air by the tail to observe symmetry in the movement of the four limbs. Scores indicate the following: 3, all four limbs extended symmetrically; 2, limbs on left side extended less or more slowly than those on the right; 1, limbs on left side showed minimal movement; and 0, forelimb on left side did not move at all.

3. Forepaw Outstretching

The rat was brought up to the edge of the table and made to walk on forelimbs while being held by the tail. Symmetry in the outstretching of both forelimbs was observed while the rat reached the table and the hindlimbs were kept in the air. Scores indicate the following: 3, both forelimbs were outstretched, and the rat walked symmetrically on forepaws; 2, left side outstretched less than the right, and forepaw walking was impaired; 1, left forelimb moved minimally; and 0, left forelimb did not move.

4. Climbing

The rat was placed on the wall of a wire cage. Normally the rat uses all four limbs to climb up the wall. When the rat was removed from the wire cage by pulling it off by the tail, the strength of attachment was noted. Scores indicate the following: 3, rat climbed easily and gripped tightly to the wire; 2, left side was impaired while climbing or did not grip as hard as the right side; and 1, rat failed to climb or tended to circle instead of climbing.

5. Body Proprioception

The rat was touched with a blunt stick on each side of the body, and the reaction to the stimulus was observed. Scores indicate the following: 3, rat reacted by turning head and was equally startled by the stimulus on both sides; 2, rat reacted slowly to stimulus on left side; and 1, rat did not respond to the stimulus placed on the left side.

6. Response to Vibrissae Touch

A blunt stick was brushed against the vibrissae on each side; the stick was moved toward the whiskers from the rear of the animal to avoid entering the visual fields. Scores indicate the following: 3, rat reacted by turning head or was equally startled by the stimulus on both sides; 2, rat reacted slowly to stimulus on left side; and 1, rat did not respond to stimulus on the left side.
